# Autonomous Graphene Vessel for Suctioning and Storing Liquid Body of Spilled Oil

**DOI:** 10.1038/srep22339

**Published:** 2016-02-29

**Authors:** Taewoo Kim, Jeong Seok Lee, Geonhui Lee, Dong Kyun Seo, Youngbin Baek, Jeyong Yoon, Seung M. Oh, Tae June Kang, Hong H. Lee, Yong Hyup Kim

**Affiliations:** 1 School of Mechanical and Aerospace Engineering, Seoul National University, Seoul 08826, South Korea; 2 School of Chemical and Biological Engineering, Seoul National University, Seoul 08826, South Korea; 3 Institute of Chemical Process, Asian Institute for Energy, Environment & Sustainability (AIEES), Seoul National University, Seoul 08826, South Korea; 4 Department of Mechanical Engineering, INHA University, Incheon 22212, South Korea

## Abstract

Despite remarkable strides in science and technology, the strategy for spilled oil collection has remained almost the same since the 1969 Santa Barbara oil spill. The graphene vessel devised here can bring about an important yet basic change in the strategy for spilled oil collection. When it is placed on the oil-covered seawater, the graphene vessel selectively separates the oil, then collects and stores the collected oil in the vessel all by itself without any external power inputs. Capillarity and gravity work together to fill this proto-type graphene vessel with the spilled oil at a rate that is higher than 20,000 liters per square meter per hour (LMH) with oil purity better than 99.9%, and allow the vessel to withstand a water head of 0.5 m. The vessel also has a superb chemical stability and recyclability. An expanded oil contact area, considerably greater than the thickness of the oil layer, forms at the reduced graphene oxide (rGO) foam interface upon contact with the spilled oil. This expanded contact area does not change much even when the oil layer thins out. As a result, the high oil collection rate is maintained throughout the recovery of spilled oil.

Oil spills have caused sea and river pollution resulting in severe environmental and ecological problems^1^,^2^,^3^. Since 1963, approximately 28,000,000 barrels of oil were spilled by accidents and the possibility of large oil spill accidents has increased with industrial development and deepwater oil drilling. Strikingly, the oil collection strategy used for the Gulf of Mexico’s 2010 spill was much the same as for the Santa Barbara’s 1969 oil spill, despite remarkable advances made in science and technology since 1969.

On the other hand, there have been a large number of studies on oil removal, either for oil absorption or oil/water separation. For oil absorption, nanomaterial sponges have extensively been used due to their low density and superhydrophobicity. Reduced graphene oxide^4^,^5^,^6^,^7^,^8^,^9^, carbon nanotube (CNT)^10^,^11^,^12^, carbon fiber^13^, and polymer^14^,^15^ were mainly utilized as a raw material for sponge fabrication, showing outstanding oil absorption capacity, efficiency, and reusability. For oil/water separation, superhydrophobic or superhydrophilic membrane and mesh, made by CNT^16^,^17^,^18^, polymer^19^,^20^,^21^, metal hydroxide^22^, or silicate^23^,^24^, were used to selectively pass oil or water for oil/water separation. In particular, membrane type^16^,^17^,^19^,^20^ was efficient for separating emulsion of oil and water due to its small pore size while mesh type^21^,^22^,^23^,^24^ yielded high flux with large pores. An oxidized copper mesh box was recently proposed for *in-situ* separation and collection of spilled oil^25^, and subsequently the mesh box was coated with palmitic acid to improve the performance^26^.

For any scheme to be useful for collecting spilled oil on seawater, a number of conditions have to be met. A floating device, when put into the seawater for the collection, has to be sturdy enough to withstand sloshing seawater and be effective even when it is tumbled over. The material used has to be chemically stable since crude oil contains organic solvents such as toluene and other hydrocarbons, which may dissolve the material. To selectively separate the oil and keep the collected oil in the device from remixing with surrounding seawater, the device walls must withstand the seawater pressure when it is submerged by the weight of collected oil and splashed by sloshing seawater.

In this work, we present an autonomous graphene vessel that satisfies the conditions. The vessel separates spilled oil from seawater, collects, and stores the collected oil in the vessel without any external power inputs. To construct the vessel, we developed an ion-mediated assembly process followed by annealing to deposit reduced graphene oxide (rGO) from a solution of graphene oxide (GO) nanoplatelets on copper mesh. This graphene vessel is essentially an enclosed empty container, the hull of which is made of copper mesh that is, in turn, coated with rGO foam covering the whole surface, inside and out. Countless pores in rGO foam quickly suction spilled oil by capillary force like sponge, and the suctioned oil flows into the vessel by gravity.

## Results

### Autonomous oil collection by graphene vessel

**Figure 1:**
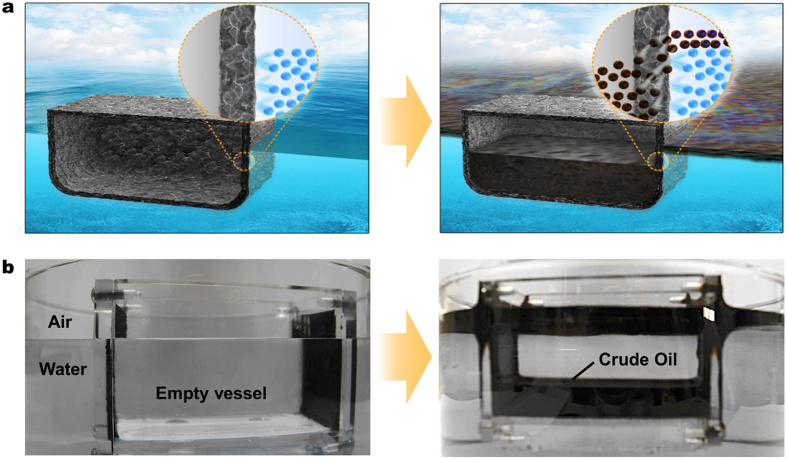
Autonomous oil collection by graphene vessel. (**a**) Schematic illustration of oil collection by graphene vessel. Graphene vessel floats on the surface of water due to its hydrophobic surface and buoyancy. Water (blue spheres) is repelled by the vessel. When the vessel gets in contact with the oil layer, it quickly absorbs oil (black spheres) by capillary force and oleophilicity of graphene foam. After the oil is fully absorbed in the foam walls of the vessel, the oil is collected into the vessel by gravity through the entire area of the vessel. (**b**) Optical images of an experimental graphene vessel collecting crude oil. Front, rear, and bottom sides of the vessel except for the two opposing sides were made of acrylic plate for observation. The vessel suctioned the crude oil floating on the water, and held the liquid body of the collected oil in the vessel.

The scheme of oil collection by the graphene vessel is illustrated in Fig. 1a. The graphene vessel is an enclosed empty container, the hull of which is made of copper mesh that is, in turn, coated with rGO foam covering the whole surface, inside and out. When the vessel is put into the seawater covered with a thin layer of spilled oil, the oil is selectively suctioned into the rGO foam by capillary force at the interface between the thin layer of oil and the foam, suctioning oil but repelling water at the interface because of the hydrophobicity and superoleophilicity^27^,^28^ of the foam (Figure S1). As the foam gets soaked with the collected oil, gravity forces the oil to flow into and fill the vessel, enabling continuous oil collection even without an external power. Because of hydrophobicity and small pores in the foam, the vessel could bear a water pressure up to 0.5 meter of water column (refer to Figure S2 and Supplementary Information).

An experimental graphene vessel, which is illustrated schematically in Fig. 1a, is shown in Fig. 1b. The second frame of the figure gives the picture that was taken after the vessel was put into a container of water covered with crude oil (Kuwait crude oil), showing that the suctioned spilled oil is held in the vessel as a liquid body. Although all sides are rendered transparent with the use of acrylic plates for observation of oil flow except for the two sides of copper mesh plates coated with rGO, no clear visualization was possible because of the blackness of the crude oil. Therefore, kerosene that was stained blue with Oil Blue N (Sigma Aldrich 391557) was used instead of crude oil, which is shown in Figure S3 and Video S1. Because of the clarity offered, demonstrations from here on are made with the stained kerosene for clear visualization.

### Fabrication of graphene vessel

**Figure 2:**
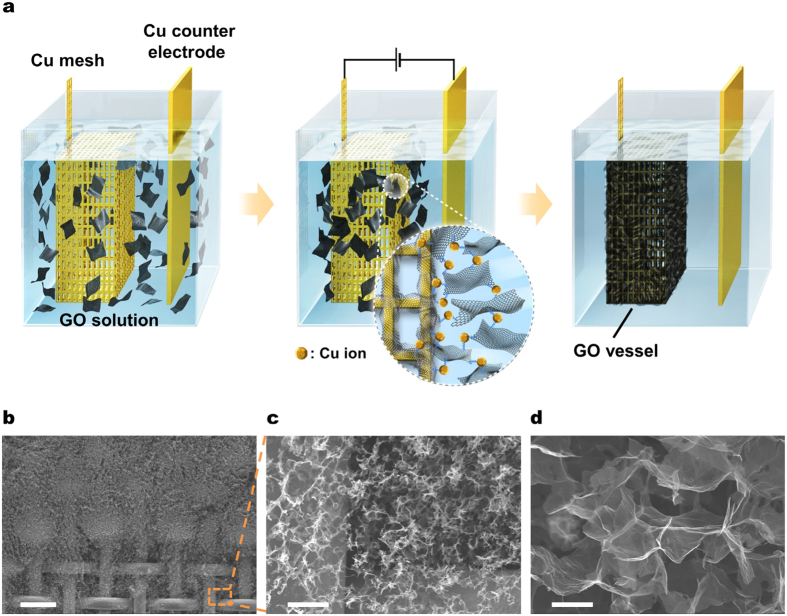
Fabrication of graphene vessel. (**a**) Schematic of ion mediated assembly (IMA) process for fabricating graphene vessel. Copper mesh container (vessel) and counter electrode are immersed in GO solution. When a dc voltage is applied between the electrodes, GO nanoplatelets are attracted to anode and an ion-mediated assembly takes place. (**b**–**d**) Scanning electron microscopy (SEM) images of rGO aerogel (foam): (**b**) Image of rGO foam at the edge of copper mesh. The foam formed uniformly on the mesh without macroscopic holes. Scale bar is 500 μm. (**c**) Magnified SEM image of the rectangular part in the image of (**b**). (**d**) Cross-sectional SEM image of the foam. The rGO nanoplatelets are interconnected to form a three-dimensional porous structure with pores in the range of several micrometers to tens of micrometers. Scale bars are 50 μm and 10 μm, respectively, in (**c**,**d**).

To construct the graphene vessel, an ion-mediated assembly (IMA) process was developed (Fig. 2a) because there was no suitable process available by which the copper mesh can be coated thick, to mm range, and uniformly all over the vessel with graphene. For the purpose, a container made of copper mesh was immersed into a tank filled with a deionized water solution of well-dispersed GO (Fig. 2a). A constant dc voltage was applied between the anode of copper mesh container and a copper plate placed in the solution acting as the cathode, which makes cupric ions dissolve from the anode. Furthermore, the GO platelets were attracted to the anode by electrostatic force. They were connected by cupric ions at the anode of copper mesh container, forming a GO hydrogel^29^. This simple procedure is all it takes to construct a GO vessel. The process is readily scalable to meter-scale due to its facile procedure. Only a larger copper mesh and enough GO solution are needed for a larger graphene wall or vessel. Because the applied voltage is not dependent on the area of graphene wall, a low voltage of 5–10 V is sufficient to make a meter-scale vessel. The process time of IMA is relatively short (10–60 sec) so that the continuous fabrication of the graphene wall is also possible.

The hydrogel was dried by freeze-drying or vacuum drying to maintain its porous structure. This aerogel was annealed in vacuum at 200 °C to reduce the GO aerogel to the rGO aerogel (Figure S4). The aerogel (foam) formed at the copper mesh edge was magnified to examine the interface between the copper mesh and the aerogel by scanning electron microscopy (SEM) (Fig. 2b). No cracks or fractures could be observed (Fig. 2c). The aerogel has a well interconnected 3-D porous network as revealed in the cross-sectional SEM image of Fig. 2d. The pore size is in the range of several micrometers to tens of micrometers.

### Oil suction characteristics of graphene vessel

**Figure 3:**
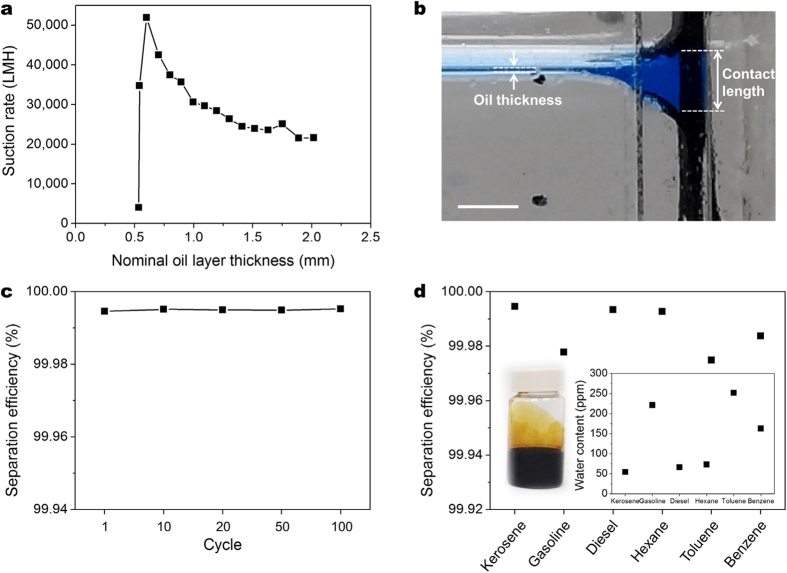
Oil suction characteristics of graphene vessel. (**a**) Dependence of oil suction rate on the thickness of oil (kerosene) layer. (**b**) Optical image of oil-water-rGO foam interface. A contact length much larger than the oil layer thickness exists at the interface because of the hydrophobicity and oleophilicity of the foam. Scale bar is 5 mm. (**c**) Recyclability of rGO vessel in terms of separation efficiency (oil purity). The efficiency is maintained better than 99.99% even after 100 cycles of usage, showing reliable reusability of the rGO vessel. (**d**) Separation efficiency of various oils and organic solvents. Gasoline, diesel, n-hexane, toluene, and 1,2-dichlorobenzene were successfully collected by the vessel showing high separation efficiencies above 99.97%. The inset graph shows water content of various oils and organic solvents. The inset figure is the optical image of crude oil collected by rGO vessel. The oil was collected by the rGO vessel dipped in crude oil for a month to check its chemical stability. Although various organic solvents, which dissolve organic compounds, are contained in crude oil, the vessel maintained its hydrophobic and oleophilic nature even after a month of direct contact with the crude oil.

With the hydrophobicity and oleophilicity of the rGO foam established (refer to Supplementary Information), the performance of the graphene vessel was evaluated with the vessel in Figure S3a. It is a rectangular vessel (8 cm × 4.2 cm with depth of 5.4 cm). Typically, a known amount of kerosene was poured into a container filled with water. Salty water having 3.5 wt% NaCl was used to mimic seawater. Figure 3a gives the suction rate or the amount of oil suctioned into and collected in the vessel in liters per square meter per hour (LMH). The rate is higher than 20,000 LMH. This rate based on the nominal oil thickness increases with decreasing kerosene layer thickness. As explained in Figure S5, the calculated nominal oil thickness for the thickness less than 0.5 mm does not represent the real oil thickness. Therefore, the suction rate around 0.5 mm does not represent the actual rate.

The oil layer thickness at the oil-foam interface was found to be considerably greater than the actual oil thickness, as shown in Fig. 3b, because of the hydrophobicity and oleophilicity of the gel. This extended contact length decreases only slightly with decreasing oil layer thickness and the ratio of the contact length to the oil layer thickness increases (Figure S6a,b). The contact length is extended due to the meniscus that forms at the oil-air-foam interface as well as the one at the oil-water-foam interface (refer to Supplementary Information). Because of almost constant contact length, the amount of oil collected per time remains relatively constant. Thus, the graphene vessel, when put into oil-spilled seawater, maintains its high oil collection rate even as the oil thins out. Maintaining a high suction rate until the oil is fully recovered is essential for oil spill accidents, which is difficult for the conventional skimmers and filtering systems to accomplish. Presence of a continuous oil film would be sufficient for ensuring the capillary action and oil collection since then the contact length would be larger than 4.6 mm, which is much larger than the effective pore diameter of the foam.

Two forces are at work for the graphene vessel: capillary force and hydrostatic pressure. The capillary force works as a driving force for oil to be suctioned into the foam. For water, on the other hand, it works as a force barring water from entering the foam, thereby enabling selective suction of oil. The hydrostatic pressure forces the oil in the foam to be permeated out of the foam and flow into the vessel (refer to Figures S7 and S8). As illustrated in Figure S9 and 10e, and Video S2, oil flows in not only from the oil-foam contact area but more importantly throughout the whole circumference of the coated foam, which enables quick collection of spilled oil.

Another performance criterion of interest is separation efficiency. The efficiency measured using Karl Fischer coulometer (Metrohm 831) was better than 99.99% in terms of oil purity. Recyclability of the vessel is of interest for prolonged use. As shown in Fig. 3c, there was almost no change in the oil purity, the oil content being in 50 ppm range (Figure S11), even after the vessel was used 100 times. Crude oil contains organic solvents such as toluene and the solvents may dissolve the material of construction for the vessel. For this reason, the separation efficiency was also measured for various oils and solvents (Fig. 3d). The vessel retained its selectively permeable property even after it had been dipped in crude oil for one month (inset figure, Fig. 3d), revealing its superb chemical stability.

### Practical operation of graphene vessel

**Figure 4:**
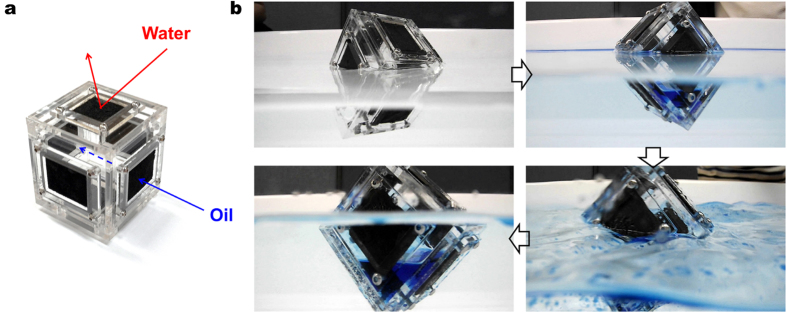
Experimental vessel for practical demonstration in wavy water. (**a**) Optical image of cube type vessel, all facets of which are coated with rGO foam with a bit of acrylic panel clearance on all sides for viewing. (**b**) The vessel was able to collect and store kerosene under high wave. Tumbling due to the wave resulted in more kerosene collected as the tumbling led to a better and more contact between kerosene/water mixture and rGO foam.

For a practical demonstration in wavy sea, a cubic vessel was fabricated to collect and hold oil under wavy condition (Fig. 4a). The copper mesh plates with rGO foams were used for all the walls of the vessel to prevent water from entering the vessel when it is tumbled over by heavy wave, with a bit of acrylic panel clearance on all sides for viewing. Even when the vessel was overturned by wave, the collected oil (kerosene) was retained in the vessel due to oil-selective permeable foam and the closed structure of the vessel (Fig. 4b and Video S3). An additional amount of kerosene was collected as the choppy water gets in contact with the foam on the closed top side. In fact, wavy water condition helps gather more oil for the graphene vessel. Therefore, when oil spill occurs in nasty weather and rough sea, to which the conventional oil containment boom and skimmer are inapplicable, the graphene vessels can be left to float on the sea to collect oil and then picked up later in nice weather.

There are organic and inorganic impurities in spilled oil. The oil near shore is often mixed with sand, pebble and seaweed. The oil in oceanic water contains floating tar balls that are blobs of petroleum. The pores of the graphene foam can be clogged by these impurities resulting in a decrease of the collection rate. However, the collected oil would cause the vessel to sink deeper into the seawater. As the vessel sinks, more fresh foam surface becomes available, and this submersion would help restore the collection rate.

## Discussion

The proto-type graphene vessel presented here, despite its simple fabrication and easy scalability, separates spilled oil almost perfectly from seawater, suctions the separated oil rapidly to the end of full recovery of the spilled oil, and stores the collected oil in the vessel by virtue of natural capillary force and gravity without any inputs from outside. These results demonstrate that man-made disaster can be remedied by natural forces and intrinsically natural material. The approach taken here for the clean-up of spilled oil, which is to rely on natural forces and to use natural materials, does provide an enlightened path for meeting scientific and technological challenges.

## Methods

### Fabrication of graphene vessel

A copper mesh (Nilaco Corp., CU-118050) with 300 μm openings was utilized to form the GO hydrogel by IMA. The mesh was cut and folded to make a mesh container. This copper mesh container was immersed into a tank filled with a deionized water solution of well-dispersed GO. The homogeneous colloidal solution was obtained by preparing GO powders via a modified Hummers method^30^ and dispersing them in deionized water with sonication. A constant dc voltage of 10 V was applied between the anode of copper mesh container and the cathode of copper plate for 1 minute by a DC power supply (ITECH, IT6720). The GO hydrogel formed on the mesh was immediately dried using a vacuum freeze dryer (Ilshin, FDS-5508) or a vacuum furnace (custom made furnace). This GO aerogel was annealed in vacuum (under 10^−2^ Torr, vacuum furnace) at 200 °C for several hours to eliminate remaining water molecules and oxygen functional groups.

### Preparation of graphene oxide

A solution mixture of graphite powder (Bay Carbon, SP-1), sulfuric acid, and potassium permanganate in a beaker was stirred for 6 hrs at 45 °C. The solution was neutralized by deionized (DI) water and hydrogen peroxide. This brown solution was subjected to dialysis to completely remove any residual acid and salt in the solution. The GO powder was obtained by filtration of the solution using an Anodisc membrane filter (47 mm diameter, 0.2 μm pore size, Whatman). The concentration of GO dispersed in DI water was 1–5 mg mL^−1^ and the solution was sonicated for 6 hrs to make a homogeneous GO suspension.

## Additional Information

**How to cite this article**: Kim, T. *et al*. Autonomous Graphene Vessel for Suctioning and Storing Liquid Body of Spilled Oil. *Sci. Rep.* **6**, 22339; doi: 10.1038/srep22339 (2016).

## Supplementary Material

Supplementary Information

Supplementary Video 1

Supplementary Video 2

Supplementary Video 3

## References

[b1] Crone T. J. & Tolstoy M. Magnitude of the 2010 Gulf of Mexico Oil Leak. Science 330, 634 (2010). 20929734 10.1126/science.1195840

[b2] Peterson C. H. . Long-Term Ecosystem Response to the Exxon Valdez Oil Spill. Science 302, 2082–2086 (2003). 14684812 10.1126/science.1084282

[b3] Bragg J. R., Prince R. C., Harner E. J. & Atlas R. M. Effectiveness of bioremediation for the Exxon Valdez oil spill. Nature 368, 413–418 (1994).

[b4] Bi H. . Highly enhanced performance of spongy graphene as an oil sorbent. J. Mater. Chem. A 2, 1652–1656 (2014).

[b5] Bi H. . Spongy Graphene as a Highly Efficient and Recyclable Sorbent for Oils and Organic Solvents. Adv. Funct. Mater. 22, 4421–4425 (2012).

[b6] Nguyen D. D., Tai N.-H., Lee S.-B. & Kuo W.-S. Superhydrophobic and superoleophilic properties of graphene-based sponges fabricated using a facile dip coating method. Energy Environ. Sci. 5, 7908–7912 (2012).

[b7] Yang S. J., Kang J. H., Jung H., Kim T. & Park C. R. Preparation of a freestanding, macroporous reduced graphene oxide film as an efficient and recyclable sorbent for oils and organic solvents. J. Mater. Chem. A 1, 9427–9432 (2013).

[b8] Zhang R. . Three-dimensional porous graphene sponges assembled with the combination of surfactant and freeze-drying. Nano Res. 7, 1477–1487 (2014).

[b9] Wu Y. . Three-dimensionally bonded spongy graphene material with super compressive elasticity and near-zero Poisson’s ratio. Nat. Commun. 6, 6141 (2015). 25601131 10.1038/ncomms7141

[b10] Gui X. . Magnetic and Highly Recyclable Macroporous Carbon Nanotubes for Spilled Oil Sorption and Separation. ACS Appl. Mater. Inter. 5, 5845–5850 (2013). 10.1021/am4015007 23721652

[b11] Gui X. . Carbon Nanotube Sponges. Adv. Mater. 22, 617–621 (2010). 20217760 10.1002/adma.200902986

[b12] Hashim D. P. . Covalently bonded three-dimensional carbon nanotube solids via boron induced nanojunctions. Sci. Rep. 2, 363 (2012). 22509463 10.1038/srep00363 PMC3325778

[b13] Bi H. . Carbon Fiber Aerogel Made from Raw Cotton: A Novel, Efficient and Recyclable Sorbent for Oils and Organic Solvents. Adv. Mater. 25, 5916–5921 (2013). 24038404 10.1002/adma.201302435

[b14] Ruan C., Ai K., Li X. & Lu L. A Superhydrophobic Sponge with Excellent Absorbency and Flame Retardancy. Angew. Chem. Int. Ed. 53, 5556–5560 (2014). 10.1002/anie.201400775 24711147

[b15] Zhu Q. . Robust superhydrophobic polyurethane sponge as a highly reusable oil-absorption material. J. Mater. Chem. A 1, 5386–5393 (2013).

[b16] Shi Z. . Ultrafast Separation of Emulsified Oil/Water Mixtures by Ultrathin Free-Standing Single-Walled Carbon Nanotube Network Films. Adv. Mater. 25, 2422–2427 (2013). 23494957 10.1002/adma.201204873

[b17] Gu J. . Robust preparation of superhydrophobic polymer/carbon nanotube hybrid membranes for highly effective removal of oils and separation of water-in-oil emulsions. J. Mater. Chem. A 2, 15268–15272 (2014).

[b18] Hu L. . Photothermal-Responsive Single-Walled Carbon Nanotube-Based Ultrathin Membranes for On/Off Switchable Separation of Oil-in-Water Nanoemulsions. ACS Nano 9, 4835–4842 (2015). 25905455 10.1021/nn5062854

[b19] Huang M. . Gravity driven separation of emulsified oil-water mixtures utilizing *in situ* polymerized superhydrophobic and superoleophilic nanofibrous membranes. J. Mater. Chem. A 1, 14071–14074 (2013).

[b20] Tao M., Xue L., Liu F. & Jiang L. An Intelligent Superwetting PVDF Membrane Showing Switchable Transport Performance for Oil/Water Separation. Adv. Mater. 26, 2943–2948 (2014). 24677285 10.1002/adma.201305112

[b21] Xue Z. . A Novel Superhydrophilic and Underwater Superoleophobic Hydrogel-Coated Mesh for Oil/Water Separation. Adv. Mater. 23, 4270–4273 (2011). 22039595 10.1002/adma.201102616

[b22] Zhang F. . Nanowire-Haired Inorganic Membranes with Superhydrophilicity and Underwater Ultralow Adhesive Superoleophobicity for High-Efficiency Oil/Water Separation. Adv. Mater. 25, 4192–4198 (2013). 23788392 10.1002/adma.201301480

[b23] Zhang L., Zhong Y., Cha D. & Wang P. A self-cleaning underwater superoleophobic mesh for oil-water separation. Sci. Rep. 3, 2326 (2013). 23900109 10.1038/srep02326 PMC3728594

[b24] Wen Q., Di J., Jiang L., Yu J. & Xu R. Zeolite-coated mesh film for efficient oil-water separation. Chem. Sci. 4, 591–595 (2013).

[b25] Wang F., Lei S., Xue M., Ou J. & Li W. *In Situ* Separation and Collection of Oil from Water Surface via a Novel Superoleophilic and Superhydrophobic Oil Containment Boom. Langmuir 30, 1281–1289 (2014). 24460039 10.1021/la403778e

[b26] Wang F. . Superhydrophobic and Superoleophilic Miniature Device for the Collection of Oils from Water Surfaces. J. Phys. Chem. C 118, 6344–6351 (2014).

[b27] Wang S., Zhang Y., Abidi N. & Cabrales L. Wettability and Surface Free Energy of Graphene Films. Langmuir 25, 11078–11081 (2009). 19735153 10.1021/la901402f

[b28] Nair R. R., Wu H. A., Jayaram P. N., Grigorieva I. V. & Geim A. K. Unimpeded Permeation of Water Through Helium-Leak–Tight Graphene-Based Membranes. Science 335, 442–444 (2012). 22282806 10.1126/science.1211694

[b29] Park S. . Graphene Oxide Papers Modified by Divalent Ions—Enhancing Mechanical Properties via Chemical Cross-Linking. ACS Nano 2, 572–578 (2008). 19206584 10.1021/nn700349a

[b30] Dikin D. A. . Preparation and characterization of graphene oxide paper. Nature 448, 457–460 (2007). 17653188 10.1038/nature06016

