# Supplementary material for: Autonomous Graphene Vessel for Suctioning and Storing Liquid Body of Spilled Oil
Source: Sci Rep. 2016 Feb 29;6:22339. doi: 10.1038/srep22339 (PMC4770310; doi:10.1038/srep22339)
Supplement: Supplementary Information [file srep22339-s1.pdf]

Supplementary Information

Autonomous Graphene Vessel for Suctioning and Storing Liquid Body of Spilled Oil

Taewoo Kim[1], Jeong Seok Lee[1], Geonhui Lee[1], Dong Kyun Seo[1], Youngbin Baek[2,3], Jeyong Yoon[2,3], Seung M. Oh[2], Tae June Kang[4*], Hong H. Lee[2*], and Yong Hyup Kim[1*]

[1]School of Mechanical and Aerospace Engineering, Seoul National University, Seoul 08826, South Korea.

[2]School of Chemical and Biological Engineering, Seoul National University, Seoul 08826, South Korea.

[3]Institute of Chemical Process, Asian Institute for Energy, Environment & Sustainability(AIEES), Seoul National University, Seoul 08826, South Korea.

[4]Department of Mechanical Engineering, INHA University, Incheon 22212, South Korea.

[*]Y. H. Kim, E-mail: yongkim@snu.ac.kr

[*]H. H. Lee, E-mail: honghlee@snu.ac.kr

[*]T. J. Kang, E-mail: tjkang@inha.ac.kr

1- Hydrophobicity and oleophilicity of rGO foam

To ascertain hydrophobicity and oleophilicity of the foam, which is needed for the selective separation and suctioning of oil from water, its wetting behavior was examined. The contact angles (CAs) of oil (kerosene) and water were measured on the foam, as shown in Figure S1a and 1b, respectively. A nearly zero CA for oil and a CA of 121° for water clearly reveal its oleophilic and hydrophobic nature of the foam. A kerosene droplet (10 μL), upon contacting the foam surface, was quickly absorbed by the foam (Figure S1c). The whole process took less than 40 ms, which is the shortest time the camera in the CA analyzer (KRÜSS, DSA100) can handle, suggesting excellent absorption rate and superoleophilicity of the foam. In contrast, a water droplet (4 μL) placed on the foam surface clung to the needle even when pressed onto the surface, implying low adhesion between the foam and water (Figure S1d). When a large water droplet (10 μL) was dropped on the surface, it bounced several times due to the hydrophobicity of the foam, showing superior water-repellent property (Figure S1e).

2- Water pressure resistance

The water pressure resistance of the foam was measured to determine the maximum water depth the foam can withstand. Salty water having 3.5 wt% NaCl (Sigma Aldrich S9888) was used to mimic sea water. Water stained by methylene blue (Sigma Aldrich M9140) was poured into a graduated cylinder, the bottom of which is in contact with a copper mesh coated with the foam. The coated copper mesh was placed on a stopper without any screen or support (refer to Figure S2). The foam endured the water pressure exerted by 0.5 m long water column.

The water pressure resistance of a porous media can be obtained by the following equation:

$$h = \frac{2\gamma \cos\theta}{\rho g R} \tag{1}$$

where γ, θ, ρ, g, R and h are the surface tension, the contact angle, the density of water, the gravitational acceleration, the effective pore radius, and the water head, respectively. With the properties of water, the effective pore radius for h of 0.5 m was calculated to be 15.1 µm.

3- Oil-foam contact length

The contact length decreased only slightly with decreasing oil layer thickness while the ratio of the contact length to the oil layer thickness increases significantly with decreasing oil thickness (Figure S6a and b). This large contact length is due to the meniscus that forms at the oil-air-foam interface as well as the one at the oil-water-foam interface. The meniscus height of oil-water-foam interface was 3 times higher than that of oil-air-foam interface due to the larger oil-water interfacial tension compared to the surface tension of oil. Furthermore, a lower contact angle of oil-water-foam interface caused by the hydrophobic and olelophilic nature of the foam resulted in a larger meniscus at oil-water-foam interface.

The contact lengths calculated by Young-Laplace equation with oil properties are in good agreement with experimental results as shown in Figure S6a. Calculated data were obtained by summing the heights of menisci at oil-air interface and oil-water interface, and the oil thickness as follows:

$$\left(\frac{\gamma_{oil}}{\rho g}\right)^{1/2} cot\,\theta_{oil/air/foam} + t_{oil} + \left(\frac{\gamma_{oil/water}}{\rho g}\right)^{1/2} cot\,\theta_{oil/water/foam} \tag{2}$$

where γ_{oil}, $\gamma_{oil/water}$, $\theta_{oil/air/rGO}$, $\theta_{oil/water/rGO}$, ρ, g, t_{oil} are the surface tension of kerosene (~28 mN/m), the kerosene-water interfacial tension (~48 mN/m), the contact angle at oil-air-foam interface, the contact angle at oil-water-foam interface, the density of kerosene, the gravitational acceleration, and the oil thickness, respectively. Contact angles were directly measured from optical images as in Figure 3b. The angles were constant in the whole range of oil thickness. The angle at the oil-air-foam interface was 56.6°±1.2° while the angle at the oil-water-foam interface was 36.2°±0.4°.

4- Oil flow path

An interesting aspect of the graphene vessel is that the foam coating the whole vessel provides in effect an expanded area for oil flow. As illustrated in Figure S9, oil flows in not only from the oil-foam contact area but more importantly throughout the whole circumference of the coated foam, which enables quick collection of spilled oil. This aspect was confirmed through a designed experiment (refer to Figure S10). The experiment showed that in the initial stage, kerosene flowed in equally throughout the whole area of foam (Figure S10b). With time, the inflow from the bottom of the vessel gradually increased due to the higher pressure difference at the lower part of the vessel (Figure S10c and d, and Video S2).

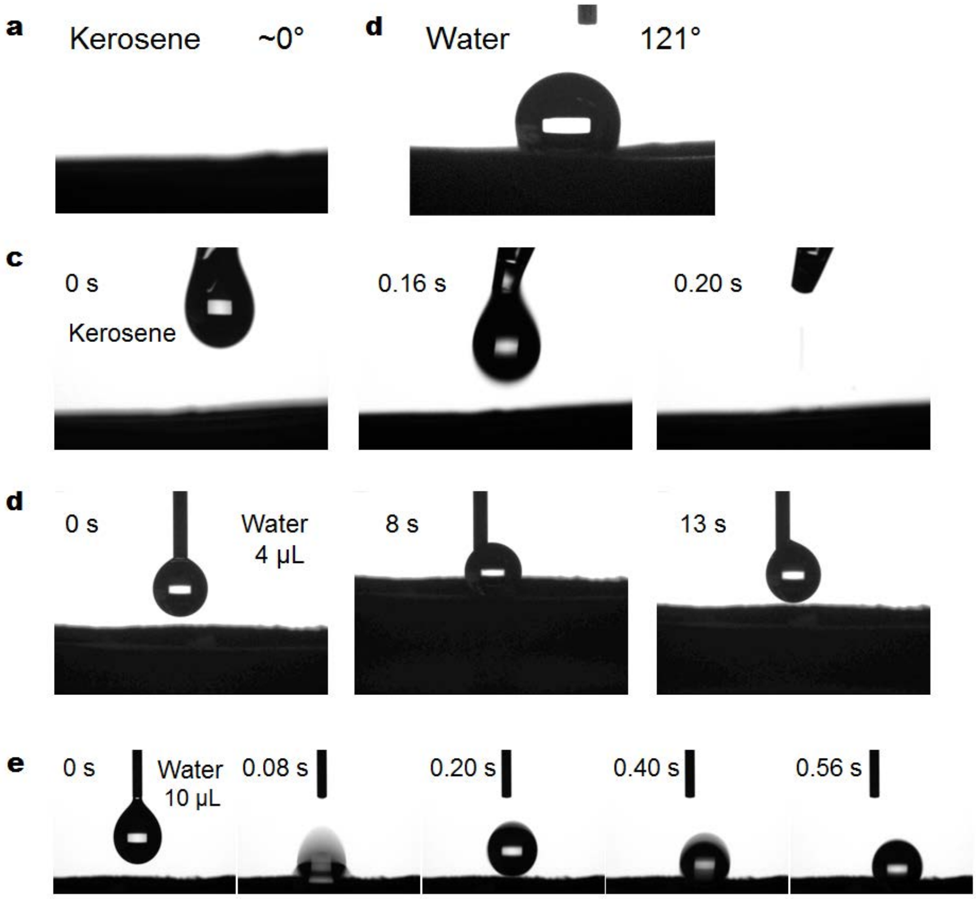

Figure S1. Images from a video contact angle (CA) device. (a) CA of oil. Oil (kerosene) was completely absorbed by rGO foam, showing almost zero CA. (b) CA of water. Water showed a CA of 121° revealing hydrophobic nature of the foam. 10 µL of water droplet was used to determine CA because a droplet smaller than the amount did not stick to the surface of the foam due to low adhesion force between water and foam. (c) Time-lapsed images of kerosene CA measurement. When a kerosene droplet contacted the foam surface, it was quickly absorbed by the foam due to the capillary force and olelophilicity of the foam. (d) Time-lapsed images of water (4 µL) CA measurement. Because of low adhesion force between water and rGO foam, water droplet could not be transferred to the surface of the foam. (e) Time-lapsed images of water (10 µL) CA measurement. The large droplet of water fell on the surface of the foam by its weight. It bounced several times due to the hydrophobicity of the foam, revealing excellent water repellent capability of the foam layer.

a

b

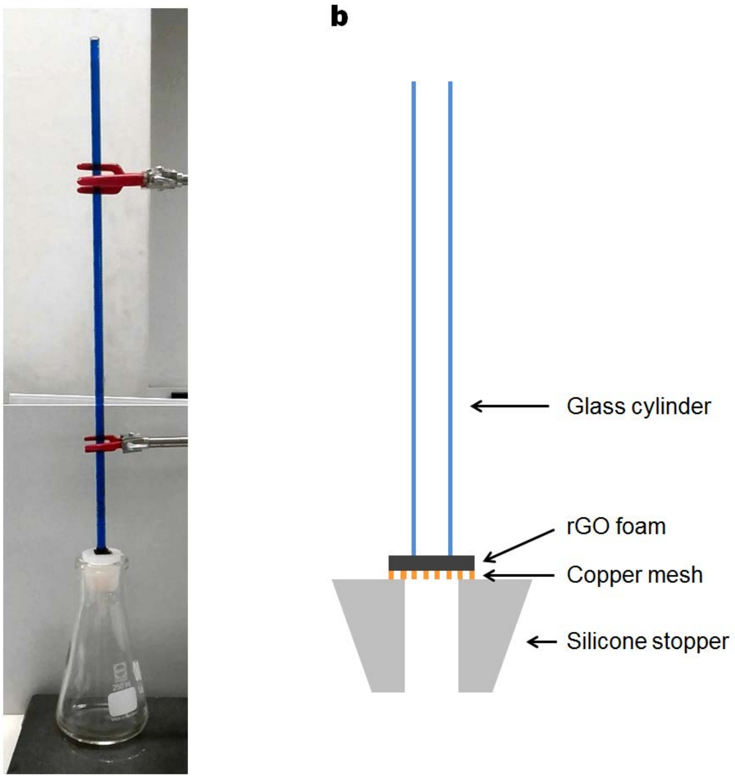

Figure S2. Measuring the pressure tolerance of rGO foam. (a) Optical image of water column (graduated cylinder) loaded above rGO foam. (b) Schematic diagram of the experimental setup.

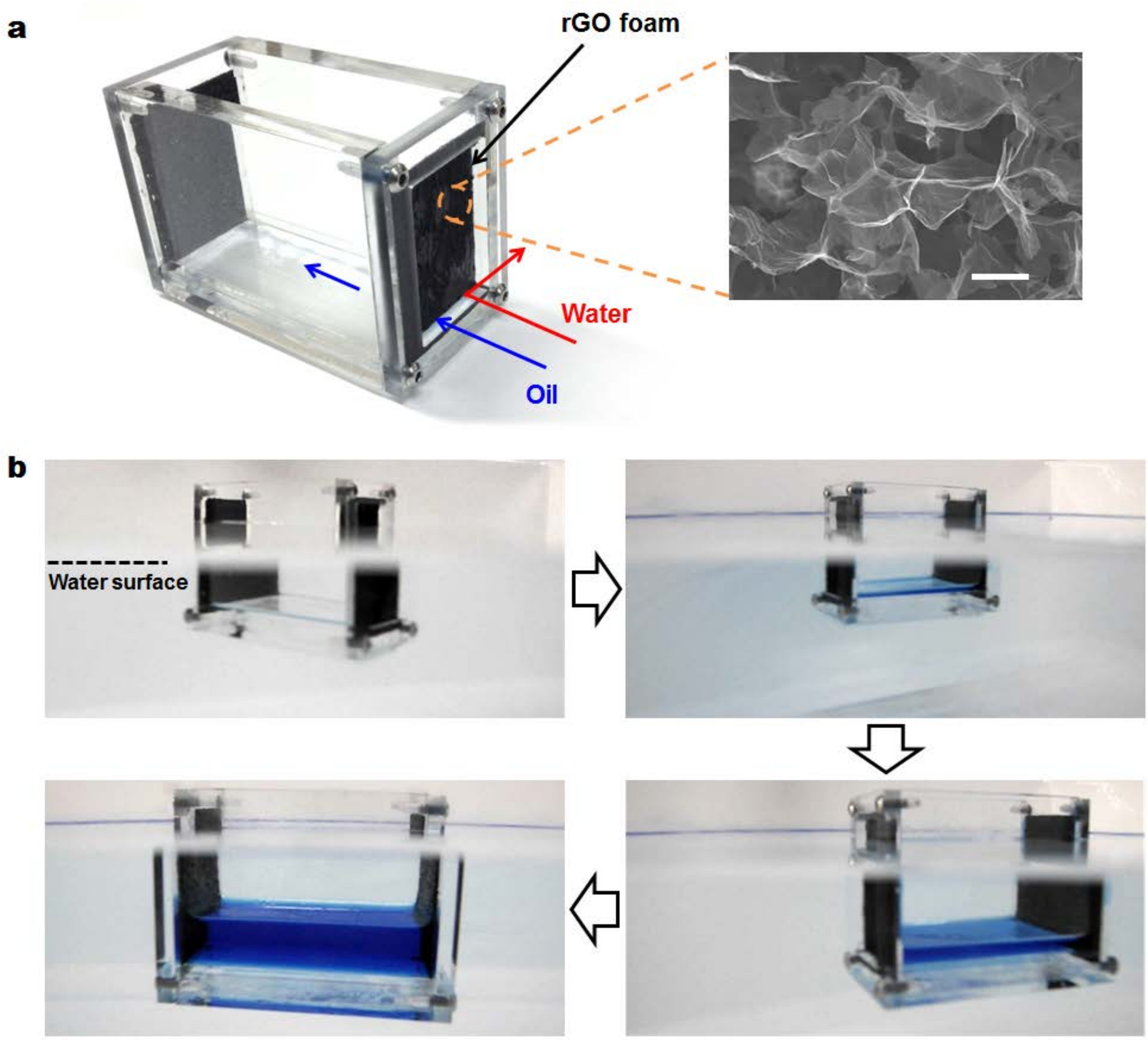

Figure S3. (a) Prototype graphene vessel fabricated with acrylic plates that were used for clear observation of oil flow. Only two sides of the vessel are the plates of copper mesh coated with rGO foam. Oil was suctioned through the two sides coated with rGO foam while water was perfectly repelled. The inset is the scanning electron microscopy (SEM) image of rGO foam, showing porous three-dimensional structure of the foam. Scale bar is 10 μm. (b) Optical images of rGO vessel floating on oil-covered water. Digital camera was installed in the boundary between water and air to observe the whole vessel. Kerosene stained with Oil Blue N was used for clear observation. The vessel freely floating in water suctioned oil and held the liquid body of the collected oil in the vessel.

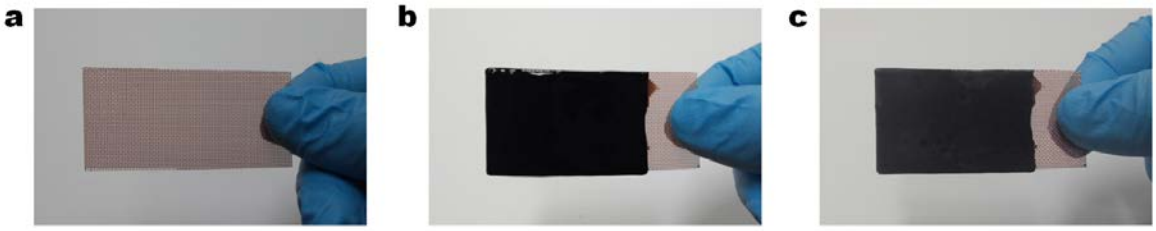

Figure S4. Optical images of (a) copper mesh, (b) copper mesh coated with GO hydrogel, and (c) copper mesh coated with rGO aerogel. The GO hydrogel is formed by the IMA process. The hydrogel is dried in vacuum to turn it into an aerogel and then annealed at 200 ^{o}C to convert it to rGO aerogel. The mesh was observed to be well coated with the aerogel without macroscopic holes.

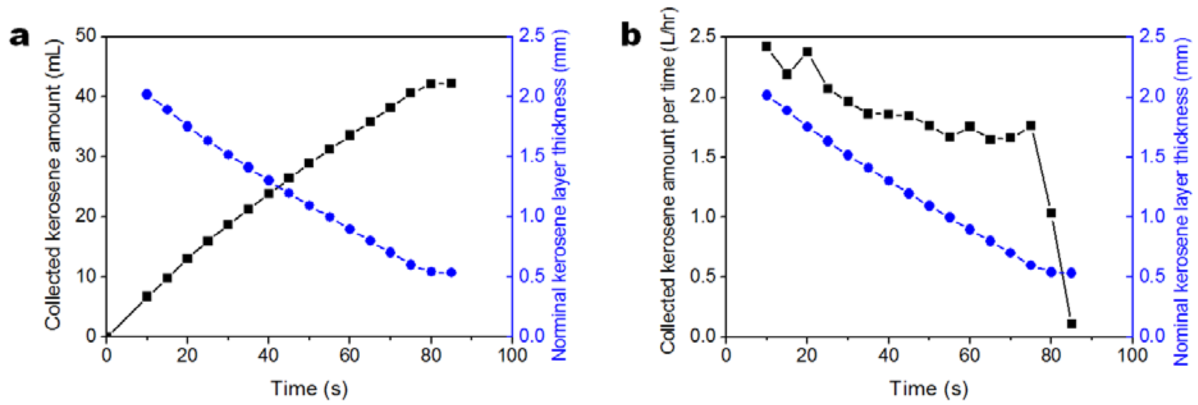

Figure S5. Measuring the pressure tolerance of rGO foam. (a) Total amount of kerosene collected in the vessel (black rectangles) and nominal kerosene layer thickness (blue circles) versus time. The amount increased linearly with time. The thickness decreased linearly with time due to the decrease in remaining kerosene on water as the oil is removed. (b) Collected kerosene amount per time (black rectangles) and nominal kerosene layer thickness (blue circles) versus time. Note that the collected kerosene amount per time remained at approximately 2 L/hr, decreasing only slightly with time despite the sharp decrease in kerosene layer thickness.

Although the nominal oil thickness that is calculated from the container cross-sectional area and the amount of remaining oil is 0.5 mm in the figure at the end of oil collection, no oil layer could be detected by naked eyes (refer to Video S1). This discrepancy is due to some oil adhering onto the container wall as the oil level recedes.

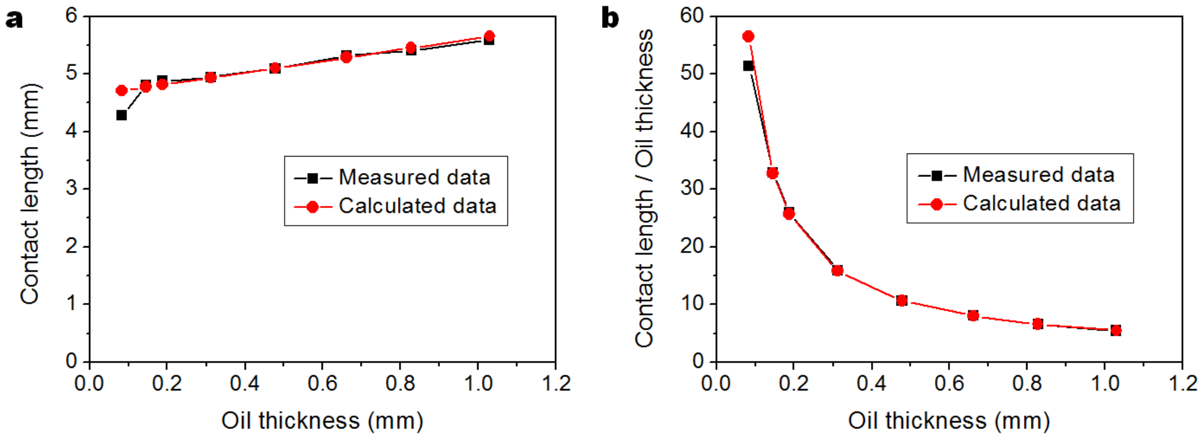

Figure S6. (a) Dependence of contact length on thickness of oil (kerosene) layer. (b) Dependence of the ratio of contact length to oil thickness on the oil thickness.

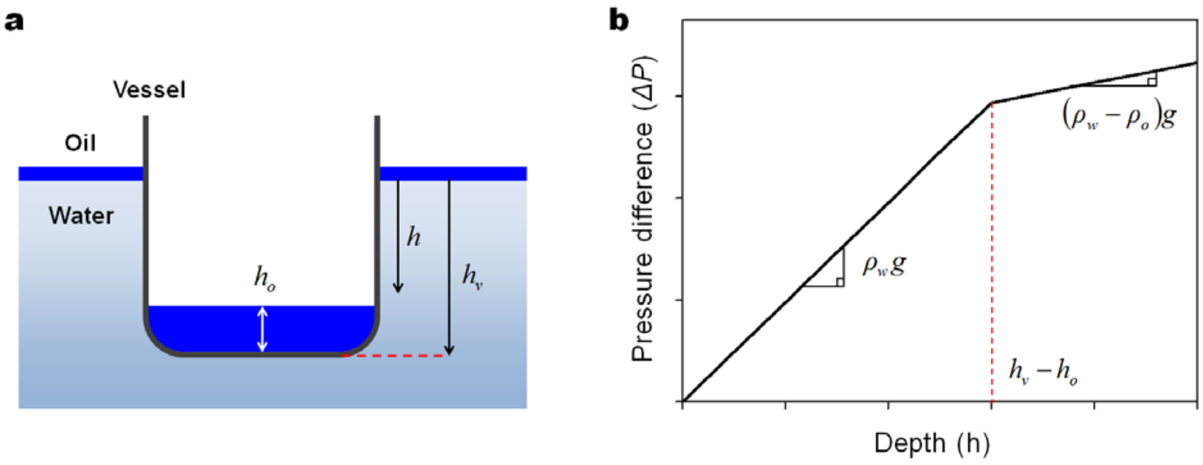

Figure S7. (a) Schematic of graphene vessel collecting oil. The symbols, h, h_v, and h_o, denote the depth of water, the depth of vessel, and the height of collected oil, respectively. (b) Pressure difference between inside and outside of the vessel as a function of water depth. Pressure difference increases with increasing water depth with the slope of $\rho_w g$ in the oil-free region and $(\rho_w - \rho_o)g$ in the oil-filled region, where ρ_w and ρ_o are the density of water and that of oil, respectively.

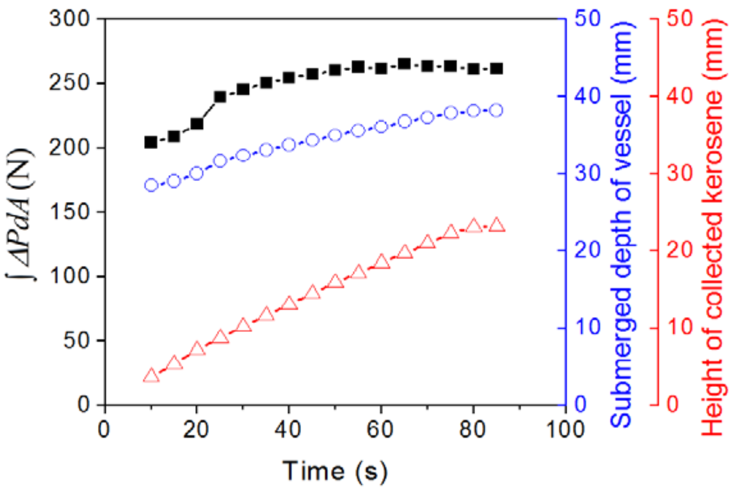

Figure S8. Integral of pressure difference, submerged depth of vessel, and height of collected kerosene versus time. The depth and height were measured from optical images, and the pressure difference was calculated using the depth and height. The hydrostatic pressure acting on the foam integrated over the area (refer to Figure S7) is given as a function of collection time. The integral of pressure difference increased about 30% with time, indicating that the increase in pressure difference due to submerged vessel is larger than the decrease in pressure difference due to collected kerosene. This increase with time or with thinning of oil layer contributed to maintaining a relatively constant suction rate with time.

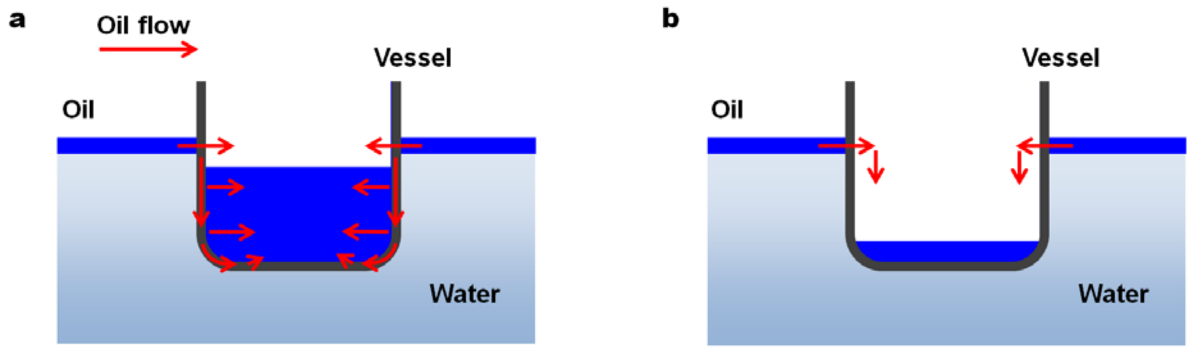

Figure S9. Schematics of oil flow when oil flows in (a) through entire area of rGO foam, (b) only through circumference of vessel that is in direct contact with oil.

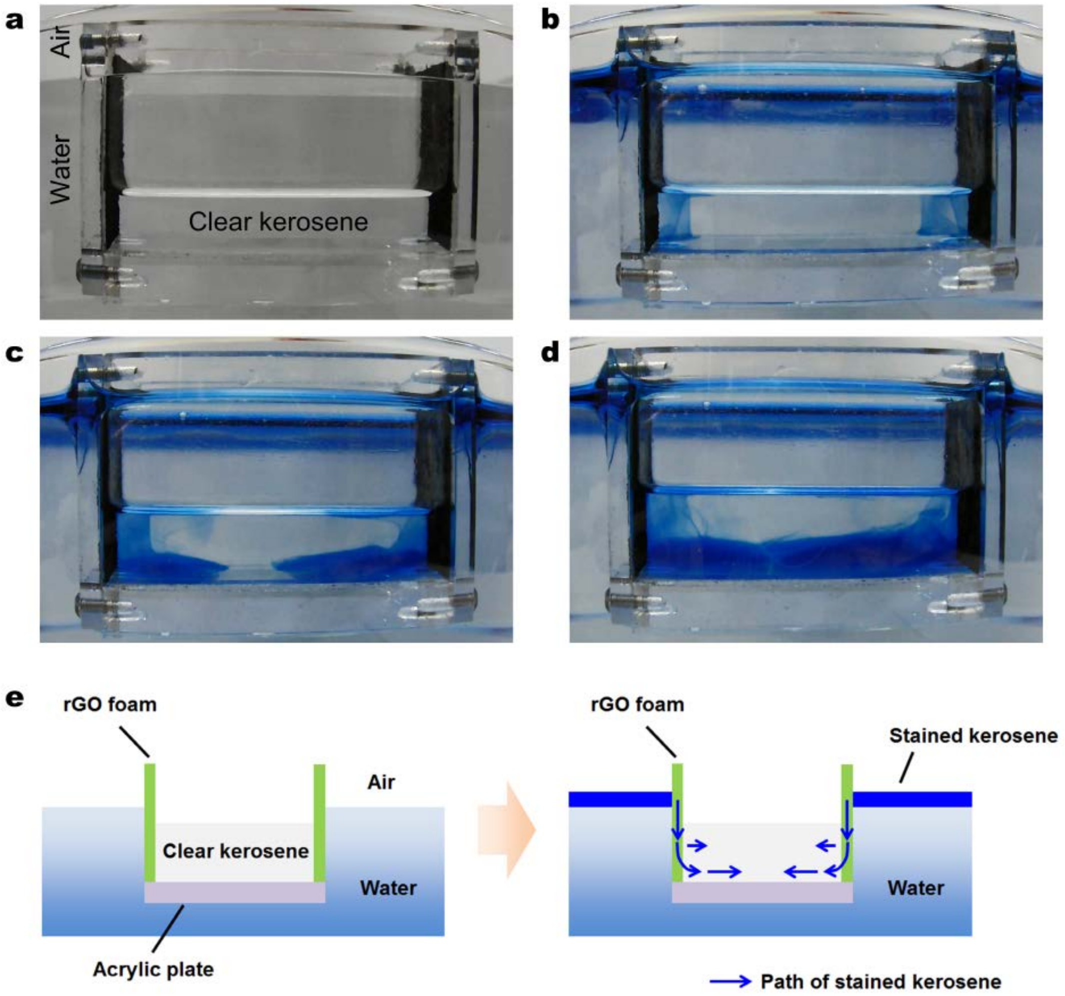

Figure S10. Visualization of oil flow during oil collection. For visualization of oil flow, the vessel surrounded by water was initially filled only with clear kerosene. Kerosene stained with Oil Blue N was then introduced to the surface of water surrounding the vessel. (a) Image of the vessel initially filled only with clear kerosene. (b) Optical image of kerosene flow after 7 sec from the time of oil introduction to the water. In the initial stage, kerosene flowed in equally throughout the whole area of foam. Optical images of kerosene flow after (c) 10 sec, (d) 30 sec following the initial flow. The inflow at the bottom of vessel gradually increased, and most of kerosene flowed in through the lower part of vessel. (e) Schematic diagrams of the experiment.

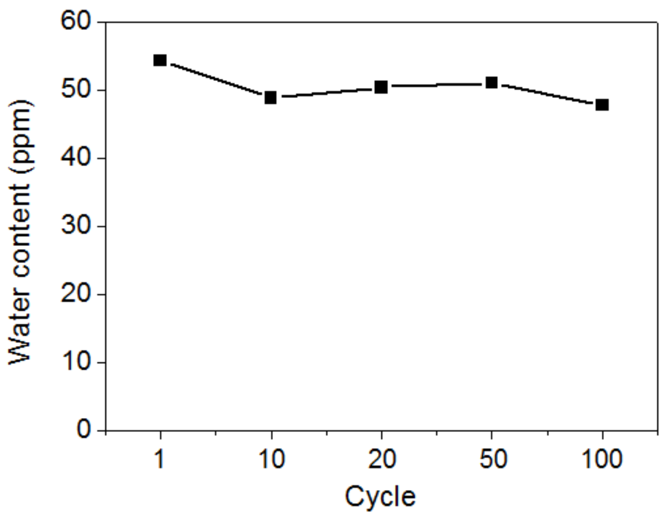

Figure S11. Recyclability of graphene vessel. Water content of kerosene as a function of the number of cycles of usage. The contents were measured by Karl Fischer coulometer.

Video legends

Video S1. rGO vessel collecting oil on water surface.

Video S2. Visualization of oil flow in rGO vessel.

Video S3. Cube type rGO vessel in wavy water.
